# Prognostic Performance of a Modified HFA-ICOS Tool for Prediction of Cardiovascular Complications in Cancer Patients

**DOI:** 10.1016/j.jacadv.2025.102344

**Published:** 2025-11-14

**Authors:** Mari Nordbø Gynnild, Victoria Vinje-Jakobsen, Joris Holtrop, Steven H.J. Hageman, Espen Holte, Geeta Gulati, Siri Lagethon Heck, Jannick A.N. Dorresteijn, Frank L.J. Visseren, Unn Merete Fagerli, Sunil Raj, Havard Dalen, Torbjørn Omland, Torgeir Wethal

**Affiliations:** a K.G. Jebsen Center for Cardiac Biomarkers, Institute of Clinical Medicine, University of Oslo, Oslo, Norway; b Clinic of Cardiology, St. Olavs University Hospital, Trondheim, Norway; c Department of Circulation and Medical Imaging, Faculty of Medicine and Health Science, NTNU – Norwegian University of Science and Technology, Trondheim, Norway; d Stroke Unit, Clinic of Medicine, St. Olavs University Hospital, Trondheim, Norway; e Department of Cardiology, Division of Medicine, Akershus University Hospital, Nordbyhagen, Norway; f Department of Vascular Medicine, University Medical Center Utrecht, Utrecht, The Netherlands; g Department of Cardiology, Division of Medicine, Oslo University Hospital, Ullevål, Oslo, Norway; h Department of Diagnostic Imaging, Akershus University, Hospital, Lørenskog, Norway; i Department of Oncology, St. Olavs University Hospital, Trondheim, Norway; j Department of Clinical and Molecular Medicine, Norwegian University of Science and Technology (NTNU), Trondheim, Norway; k Department of Medicine, Levanger Hospital, Nord-Trøndelag Hospital Trust, Levanger, Norway; l Department of Neuromedicine and Movement Science, Faculty of Medicine and Health Science, NTNU – Norwegian University of Science and Technology, Trondheim, Norway

**Keywords:** cancer, cardio-oncology, cardiovascular risk, HFA-ICOS risk stratification tool, prognosis, risk assessment, risk stratification

## Abstract

**Background:**

The 2022 European Society of Cardiology Cardio-oncology Guidelines recommend the Heart Failure Association and International Cardio-Oncology Society risk stratification tool to predict cardiovascular disease (CVD) risk in cancer patients. Developed from expert consensus rather than mathematical derivation, its quantitative prognostic value in unselected cancer populations remains uncertain.

**Objectives:**

The objective of the study was to assess the prognostic utility of a modified Heart Failure Association and International Cardio-Oncology Society tool, excluding biomarkers and imaging, in predicting CVD complications in cancer patients, thereby providing a quantitative correlate to an expert consensus-based method.

**Methods:**

We included 2,290 cancer patients from the third follow-up of the Trøndelag Health study, diagnosed within 4 years of participation, and followed through 2023. Cancer therapy details were obtained from the Norwegian Cancer Registry. The primary outcome was a composite of myocardial infarction, heart failure hospitalization, stroke, or CVD mortality, identified via national registries.

**Results:**

The mean age was 65.8 years; 48% were female. The most common cancers were gastrointestinal (23%), prostate (19%), and breast (12%). The median follow-up was 6.9 years. The modified tool classified 28% as low-, 35% moderate-, 34% high-, and 3% very high-risk. CVD incidence rose across categories: 7%, 21%, 29%, and 37% (*P* < 0.001). The C-statistic was 0.696 (95% CI: 0.674-0.718), and 0.746 (95% CI: 0.676-0.812) among anthracycline-treated patients. Within the low-to-moderate group, event rates ranged from 6% to 8% (0-1 points) to 24% to 27% (3-4 points).

**Conclusions:**

The tool stratifies CVD risk in cancer patients and may guide tailored follow-up and cardioprotection. Heterogeneity within moderate-risk patients highlights the need for refinement to improve individualized assessment.

Cancer therapy-related toxicity and cardiovascular complications due to cancer and cancer therapy is a growing medical problem.^1^ Baseline cardiovascular disease (CVD) risk assessment is essential for identifying patients who may need intensified follow-up and cardioprotective treatment.^1^^,^^2^ However, CVD risk stratification tools specifically for cancer patients are limited and not extensively validated.^2^^,^^3^

The 2022 European Society of Cardiology guidelines on cardio-oncology recommend using the HFA-ICOS (Heart Failure Association and the International Cardio-Oncology Society) risk stratification tool to predict the risk of heart failure (HF) and other CVD complications during and after cancer therapy.^1^ This tool categorizes patients into risk categories based on literature review and expert consensus,^3^ contrary to other models mathematically derived from data and validated in external cohorts.^4^^,^^5^ However, the tools’ design accounts for the complexity and variability of cancer treatments and their wide-ranging cardiovascular impacts,^1^^,^^3^^,^^6^ which traditional CVD models are lacking.

So far, validation of the HFA-ICOS tool has been restricted to patients with breast cancer,^7^, ^8^, ^9^, ^10^ melanoma,^11^ and chronic myeloid leukemia,^12^ and focused on 7 specific cancer treatments. The actual risk associated with each risk category remains unknown, and validation of the tool has been highlighted as a key research priority.^1^^,^^2^ Accordingly, studies are needed to confirm that this consensus-based stratification appropriately separates patients into higher- and lower-risk groups in real-world data, and to establish a quantitative correlation between assigned risk strata and subsequent clinical outcomes. Based on the predictors included, we hypothesized that the tool may be useful across a broader oncological population, regardless of the cancer type or treatment. Therefore, this study aimed to assess the prognostic performance of a modified HFA-ICOS tool in a large, general cancer population, and in subgroups receiving anthracyclines, human epidermal growth factor receptor 2 (HER2)-targeted therapy, vascular endothelial growth factor (VEGF) inhibitors and myeloma therapy.

## Methods

### Study design and population

**Figure 1:**
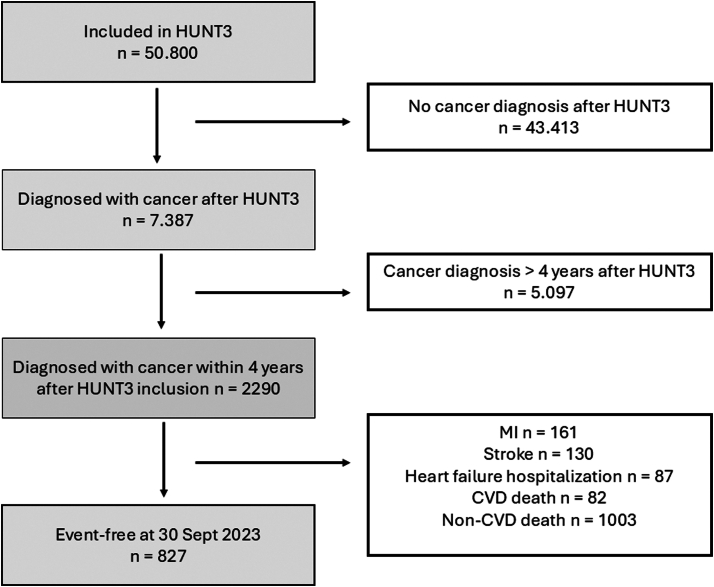
**Flowchart of Participants** Flow diagram of study participants showing inclusion and exclusion criteria. Events represent only the first occurrence per individual. CVD = cardiovascular disease; HUNT = the Trøndelag Health Study; MI = myocardial infarction.

This study included 2,290 participants from the third follow-up of the Trøndelag Health study (HUNT3), conducted between 2006 and 2008 (Figure 1). Participants diagnosed with cancer within 4 years after inclusion in HUNT3 were eligible for analysis (2006-2012) and were followed for outcomes through December 2023 (Supplemental Figure 1). The HUNT study is a longitudinal population-based health study in mid-Norway that invited all adult residents to participate. Data collected included demographics, clinical data, questionnaires, and blood tests. Further details are described elsewhere.^13^ The Regional Committee for Medical and Health Research Ethics approved the study (REC 590420) and written informed consent was obtained from all participants.

### Data collection and definition of baseline variables

CVD risk stratification was based on HUNT3 data, supplemented by diagnostic codes from the local hospital registry (Supplemental Table 1). Hypertension was defined as the use of antihypertensive drugs or blood pressure >140/90 mm Hg. Diabetes mellitus was identified through self-report or hospital records. Chronic kidney disease (CKD) was defined as estimated glomerular filtration rate <60 mL/min/1.73 m^2^ or CKD diagnosis.

Linkage to the Norwegian Cancer Registry, which has a coverage of 98% to 99% since the early 2000s, provided information about tumor characteristics (stage, grade and hormone receptor status, and previous cancer history from 1953 onward) and treatment.^14^ Data on systemic anticancer therapies administered intravenously in hospitals (available from 2003 onward) were obtained through the INSPIRE (INcreaSe PharmaceutIcal REporting) project, which extracts information from hospital software systems (CSAM Cytodose, CSAM Health Group AS) used across Central Norway Regional Health Trusts.^15^ Potential cardiotoxic radiotherapy (RT) was defined as radiation directed toward breast, mediastinum or thoracic region.

### The modified HFA-ICOS cardiovascular toxicity risk stratification

All 2,290 participants were divided into 4 risk categories (low, moderate, high, and very high) using a modified version of the HFA-ICOS risk assessment tool. The original tool provides treatment-specific risk stratification for 7 cardiotoxic cancer therapies^3^: anthracyclines, HER2-targeted therapies, VEGF inhibitors, breakpoint cluster region–Abelson oncogene locus inhibitors, multiple myeloma therapies, rapidly accelerated fibrosarcoma, and mitogen-activated protein kinase inhibitors. Many cancers may involve one or more of the relevant therapies (particularly anthracyclines and VEGF inhibitors) and could therefore be eligible for risk stratification according to the specific HFA-ICOS risk stratification schemes.^1^ Several of these agents (eg, rapidly accelerated fibrosarcoma/mitogen-activated protein kinase and VEGF inhibitors) were not yet in use during the study period, and oral agents were not captured (ie, breakpoint cluster region–Abelson oncogene locus inhibitors). In addition, electrocardiograms, echocardiography, and cardiac biomarkers (troponin, N-terminal pro-B-type natriuretic peptide) were unavailable. Table 1 provides a complete overview of original HFA-ICOS predictors and availability in our cohort.

**Table 1:** Assignment of Risk Levels Based on a Modified HFA-ICOS Tool

Predictor (Original HFA-ICOS)	Assigned Risk^a^
Heart failure or cardiomyopathy	Very high
Arterial vascular disease (PAD, TIA, stroke)	High
Myocardial infarction or previous coronary revascularization (PCI or CABG)	High
Stable angina	High
Previous anthracycline exposure	High
Age ≥80 years	High
Age 65–79 years	Medium 2p
Arrhythmia^b^	Medium 2p
Hypertension	Medium 1p
Diabetes mellitus	Medium 1p
Hyperlipidemia^c^	Medium 1p
Chronic kidney disease	Medium 1p
Current smoker	Medium 1p
Obesity (body mass index >30 kg/m^2^)	Medium 1p
Prior radiotherapy to left chest or mediastinum	Medium 1p
Previous nonanthracycline-based chemotherapy	Medium 1p
Elevated baseline cardiac troponin	Information not available
Elevated baseline NT-proBNP	Information not available
Left ventricular ejection fraction <50%	Information not available

CABG = coronary artery bypass grafting; HFA-ICOS = Heart Failure Association and International Cardio-Oncology Society; HUNT = the Trøndelag Health Study; NT-proBNP = N-terminal pro–B-type natriuretic peptide; p = points; PAD = peripheral artery disease; PCI = percutaneous coronary intervention; TIA = transient ischemic attack.

a Patients were categorized as follows: low risk, no risk factors or 1 medium-level factor (1 point); moderate risk, 2 to 4 medium-level risk points; high risk, ≥5 medium-level points or any high-risk factor; and very high risk, presence of any very-high-risk factor.

b Atrial fibrillation, atrial flutter, ventricular tachycardia.

c Nonhigh-density lipoprotein cholesterol >3.8 mmol/L. Details in Supplemental Table 2.

Given these limitations, and in line with European Society of Cardiology guideline recommendations for baseline CVD risk assessment in all cancer patients, we applied a pragmatic, harmonized scoring approach using available clinical predictors. The available predictors included age, hypertension, diabetes mellitus, CKD, smoking, obesity, prior CVD, and prior cardiotoxic cancer treatment. For available predictors, point assignment was consistent across the different HFA-ICOS stratification schemes, with a few exceptions. Where risk weighting differed between treatment-specific schemes, the most frequently assigned risk level was used for consistency. Patients were categorized as follows: low risk, no risk factors or one medium-level factor (1 point); moderate risk, 2 to 4 medium-level risk points; high risk, ≥5 medium-level points or any high-risk factor; and very high risk, presence of any very high-risk factor. Further details on the adapted scoring are provided in Supplemental Table 2 and Supplemental Methods. Subgroup analyses were performed in patients receiving therapies for which the HFA-ICOS tool was originally developed: 1) anthracyclines alone; and 2) pooled group receiving anthracyclines, HER2-targeted therapy, VEGF inhibitors, or myeloma treatment. For these, the high- and very high-risk categories were combined due to sample sizes, and consistency in recommendations across these categories.

### Cardiovascular outcomes

Follow-up started at cancer diagnosis and continued through September 2023. CVD complications included the first occurrence of HF hospitalization (primary diagnosis), nonfatal stroke (primary diagnosis), nonfatal myocardial infarction (MI) (primary and secondary diagnosis) or CVD death (primary cause of death). Diagnostic codes are listed in Supplemental Table 1. Secondary endpoints were HF hospitalization and all-cause mortality. Outcomes were obtained via linkage to hospital registries and the Norwegian Cause of Death Registry.

### Statistical analysis

Descriptive data are presented as mean (SD), median (IQR) or proportions, as appropriate. Baseline characteristics were stratified by HFA-ICOS risk category. To visualize trends in event rates, we calculated the interval incidence per 6 months. We present Kaplan-Meier survival curves for the 4 risk categories and the risk of the combined endpoint of HF hospitalization, stroke, MI, or CVD death, as well as for HF hospitalization alone and all-cause mortality alone. Competing risk analysis was performed using cumulative incidence functions. Discriminative abilities were assessed using the Harrel C-statistic. Calibration accuracy could not be assessed, as the original HFA-ICOS tool provides no published risk equation or expected probabilities; therefore, only discrimination (C-statistics) is reported. All event frequencies are presented as cumulative incidences over the entire follow-up period until December 2023, with median follow-up times provided for each analysis.

To evaluate the association between individual HFA-ICOS predictors and CVD complications, a Fine and Gray regression model was used to generate unadjusted and age- and sex-adjusted subdistribution HRs (sHRs) with 95% CIs. Predictors included HF, valvular heart disease, coronary artery disease, cerebrovascular disease, arrhythmia, age (continuous and categorical [≥80, 65-79, <65 years]), hypertension, systolic blood pressure (continuous), diabetes mellitus, CKD, estimated glomerular filtration rate (continuous), prior chemotherapy, anthracycline and trastuzumab exposure, left chest or mediastinal RT, smoking, and obesity (body mass index [BMI] > 30 kg/m^2^ or continuous BMI).

We performed sensitivity analyses excluding participants with metastatic disease and supplementary analyses in those surviving the first 2 years. Missing data (1% to 4% per predictor) were imputed using the MICE package in R (R Foundation for Statistical Computing). To handle missing values across different variable types, predictive mean matching, logistic regression, and polytomous regression were applied as imputation methods. A random sample of the data set was selected for imputation, and the process included 5 iterations across 5 imputed data sets (m = 5). A single imputed data set was randomly selected from the imputed sets. The outcome was included using a Nelson Aalen estimator. Convergence was assessed visually. All statistical analyses were performed using R statistical software (version 4.1.3) (packages *survival, survminer, Hmisc, cmprsk*).

## Results

### Baseline characteristics

Among 2,290 participants, the mean age was 65.8 (SD 12.4) years, and 48% were female. CVD risk factors are shown in Table 2. The most common cancer types were gastrointestinal (23%), prostate (19%), and breast (12%), whereas 8% had hematological malignancies. At diagnosis, 19% had metastatic disease. Using the modified HFA-ICOS tool, 631 (28%) were assigned to the low-risk category, 806 (35%) to moderate, 785 (34%) to high-risk, and 68 (3%) to the very high-risk category. Established CVD was present in 67% and 100% of those in the high and very high-risk categories, respectively.

**Table 2:** Baseline Characteristics Stratified by HFA-ICOS Risk Group

	Total (N = 2,290)	Low Risk (n = 631)	Moderate Risk (n = 806)	High Risk (n = 785)	Very High Risk (n = 68)
Age, y	65.8 (12.4)	52.6 (9.2)	66.9 (8.6)	74.4 (8.9)	73.9 (8.5)
Male	1,194 (52%)	279 (44%)	418 (52%)	453 (58%)	44 (65%)
Diabetes mellitus	186 (8%)	5 (1%)	46 (6%)	119 (15%)	16 (24%)
Coronary artery disease	382 (17%)	0%	0%	338 (43%)	44 (65%)
Cerebrovascular disease	200 (9%)	0%	0%	188 (24%)	12 (18%)
Peripheral artery disease	146 (6%)	0%	0%	130 (17%)	16 (24%)
Heart failure	68 (3%)	0%	0%	0%	68 (100%)
Valvular disease	92 (4%)	0%	0%	75 (10%)	17 (25%)
Atrial fibrillation	186 (9%)	0%	44 (5%)	103 (13%)	39 (57%)
Chronic kidney disease^a^	245 (11%)	0%	33 (4%)	183 (23%)	29 (43%)
Smoking	556 (24%)	137 (22%)	223 (29%)	185 (25%)	11 (18%)
Body mass index >30 kg/m^2^	568 (25%)	56 (9%)	255 (32%)	233 (30%)	24 (35%)
Systolic blood pressure (mm Hg)	137 (20)	127 (16)	142 (19)	141 (20)	133 (21)
Use of antihypertensives	882 (39%)	52 (8%)	298 (37%)	483 (62%)	49 (72%)
Total cholesterol (mmol/L)	5.6 (1.1)	5.6 (1.0)	5.8 (1.1)	5.4 (1.2)	4.9 (1.1)
HDL cholesterol (mmol/L)	1.3 (0.4)	1.4 (0.3)	1.4 (0.4)	1.3 (0.3)	1.2 (0.3)
LDL cholesterol (mmol/L)^b^	3.9 (1.0)	4.0 (1.0)	4.1 (1.0)	3.7 (1.1)	3.4 (1.0)
Cancer diagnosis					
Gastrointestinal cancer	529 (23%)	103 (16%)	177 (22%)	223 (28%)	26 (38%)
Lung cancer	207 (9%)	35 (6%)	66 (8%)	99 (13%)	7 (10%)
Melanoma	272 (12%)	84 (13%)	87 (11%)	93 (12%)	8 (12%)
Breast cancer	273 (12%)	130 (21%)	93 (12%)	44 (6%)	6 (9%)
Female genital cancer	133 (6%)	52 (8%)	47 (6%)	31 (4%)	3 (4%)
Prostate cancer	425 (19%)	111 (18%)	162 (20%)	142 (18%)	10 (15%)
Lymphoma or hematological cancer	179 (8%)	47 (7%)	62 (8%)	63 (8%)	7 (10%)
Other solid tumors^c^	272 (12%)	69 (11%)	112 (14%)	90 (12%)	1 (1%)
Metastatic disease^d^	429 (19%)	85 (13%)	141 (17%)	188 (24%)	15 (22%)

Values are mean (SD) or n (proportion).

HDL = high-density lipoprotein; LDL = low-density lipoprotein; other abbreviations as in Table 1.

a Estimated glomerular filtration rate (GFR) < 60.

b Calculated by the Friedewald formula.

c Cancer of lip, oral cavity and pharynx, bone cancer, mesothelial or soft tissue cancer, central nervous system cancer, testis cancer, or endocrine cancer.

d Distant metastatic disease according to SEER (U.S. National Cancer Institute's Surveillance, Epidemiology and End Results) Program. Calculated and directly measured LDL-C have shown good agreement.^16^ Definitions are described in Supplemental Table 1.

Initial cancer treatment included chemotherapy in 35%, RT in 31%, and surgery in 66% (Table 3). Detailed characteristics and chemotherapeutic regimens are shown in Supplemental Tables 3 to 5. Overall, 255 (11%) received anthracyclines, 29 (1%) received HER2-targeted therapy, 52 (2%) received an intravenous VEGF inhibitor, 41 (2%) received myeloma therapy, and 157 (7%) received cardiotoxic RT.

**Table 3:** Cancer Therapy Received After the Baseline Risk Assessment

	Total (N = 2,290)	Low Risk (n = 631)	Moderate Risk (n = 806)	High Risk (n = 785)	Very High Risk (n = 68)
Chemotherapy (total)	796 (35%)	300 (48%)	301 (37%)	182 (23%)	13 (19%)
Anthracyclines	255 (11%)	127 (20%)	94 (12%)	31 (4%)	3 (4%)
Anti-HER2 treatment	29 (1%)	21 (3%)	7 (1%)	0%	1 (1%)
Intravenous VEGF inhibitors	52 (2%)	23 (4%)	19 (2%)	10 (1%)	0 (0%)
Myeloma treatment	41 (2%)	7 (1%)	12 (1%)	18 (2%)	4 (6%)
RT	761 (31%)	278 (44%)	271 (34%)	196 (25%)	16 (24%)
Left breast or mediastinal RT	157 (7%)	64 (10%)	60 (7%)	49 (6%)	4 (6%)
Surgery	1,520 (66%)	484 (77%)	564 (70%)	441 (56%)	31 (46%)

HER2 = Human Epidermal Growth factor receptor 2; RT = radiotherapy; VEGF = vascular endothelial growth factor.

### Adverse cardiovascular outcomes

**Figure 2:**
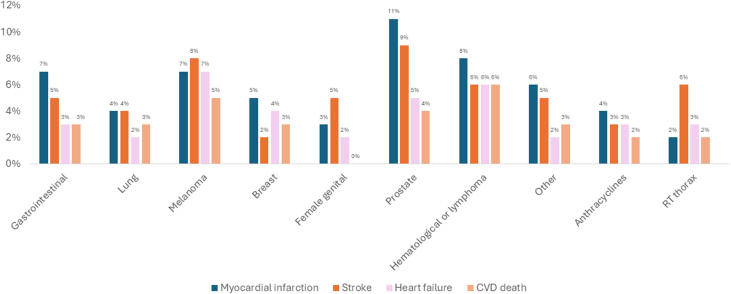
**Cardiovascular Complications by Cancer Type** Distribution of cardiovascular complications stratified by cancer type. RT = radiotherapy; other abbreviations as in Figure 1.

During a median follow-up of 6.9 (1.4-12.2) years, 460 participants experienced a (first) CVD event; 87 HF hospitalizations (19%), 161 MIs (35%), 130 strokes (28%), and 82 CVD deaths (18%). In addition, 1,003 patients (44%) died from non-CVD causes, of whom 85% had cancer as the primary cause. Overall, 435 deaths occurred within the first year after cancer diagnosis and 91 CVD events (20%) occurred within this timeframe (Supplemental Figure 2). CVD complications by cancer type are shown in Figure 2. Considering HF hospitalization alone, 116 (5%) participants were hospitalized for HF as the first event over a median follow-up of 8.3 (1.6-12.6) years.

In total, 1,335 (58%) participants died during the follow-up. Among them, 947 (71%) died from cancer, with a median time to death of 1.5 (0.4-4.3) years. CVD death occurred in 167 (7%) patients and remaining deaths were due to other causes. Supplemental Table 6 shows first events per HFA-ICOS risk category.

### Performance of the modified HFA-ICOS risk assessment tool

**Figure 3:**
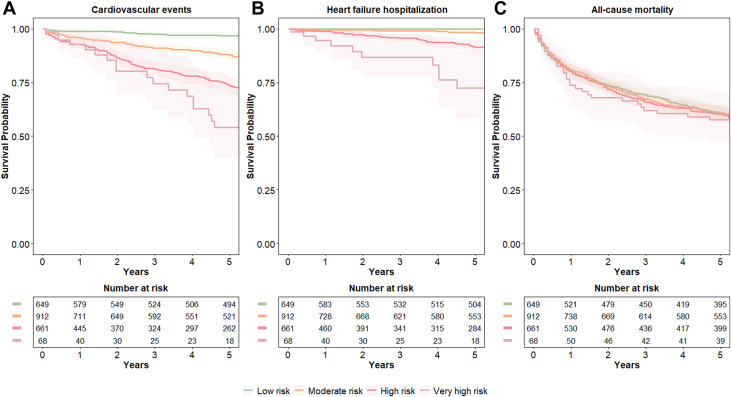
**Event-free Survival by Risk Category** Survival curves showing (A) event-free survival for cardiovascular events, (B) heart failure hospitalization, and (C) all-cause mortality, stratified by HFA-ICOS risk category. Shaded areas represent 95% CIs.

CVD incidence during the follow-up (median 6.9 [1.4-12.2] years) increased progressively across the HFA-ICOS risk categories (*P* value <0.001): 7% in low, 21% in moderate, 29% in high, and 37% in the very high-risk category (Figure 3A, Central Illustration). The C-statistic for predicting CVD events was 0.696 (95% CI: 0.674-0.718). Relative to the low-risk category, HRs were 1.38 (95% CI: 1.04-1.72) for moderate, 2.16 (95% CI: 1.83-2.49) for high, and 2.63 (95% CI: 2.13-3.12) for very high risk.

**Central Illustration:**
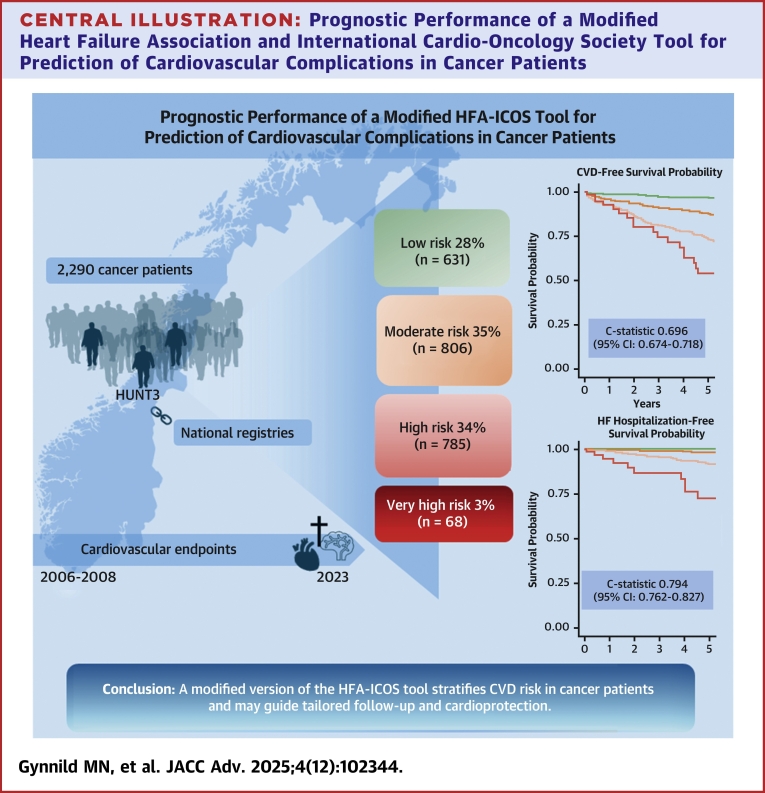
**Prognostic Performance of a Modified Heart Failure Association and International Cardio-Oncology Society Tool for Prediction of Cardiovascular Complications in Cancer Patients** HFA-ICOS = Heart Failure Association and International Cardio-Oncology Society.

**Figure 4:**
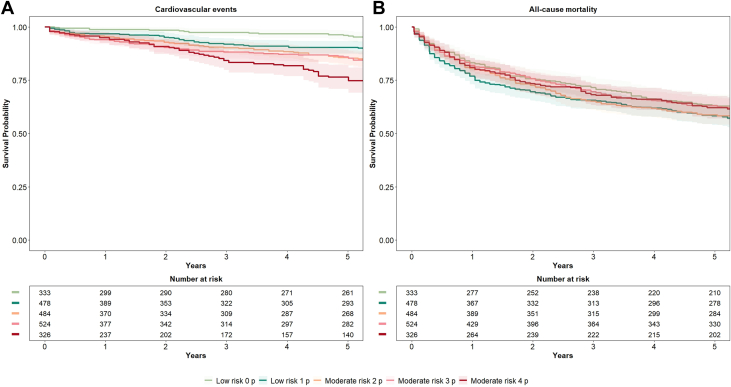
**Survival by Heart Failure Association and International Cardio-Oncology Society Point Score** Event-free survival curves for (A) cardiovascular events and (B) all-cause mortality, by points assigned within the HFA-ICOS low and moderate risk groups. Shaded areas represent 95% CIs.

Among patients classified as low or moderate risk, event incidence over follow-up increased by points assigned (Figure 4); patients with 0 points had a 6% incidence rate, 1 point had 8%, 2 points had 13%, 3 points had 24%, whereas those with 4 points had a 27% incidence rate. The C-statistic within the low-to-moderate risk category was 0.645 (95% CI: 0.618-0.673). Competing risk-adjusted cumulative incidences per risk category are shown in Supplemental Figure 3. When predicting the risk of CVD events in patients classified as low-moderate risk vs high-very high risk, HFA-ICOS had a sensitivity of 55%, a specificity of 67%, a positive predictive value of 30%, a negative predictive value of 86%, and a global accuracy of 65%.

When assessing HF hospitalizations alone as outcome, the risk of HF was higher in the high- and very high-risk categories (0% in the low-risk, 4% in the moderate, 9% in high, and 21% in the very high-risk category) (C-statistics 0.794 [95% CI: 0.762-0.827]), Figure 3B. All-cause mortality increased with HFA-ICOS risk category: 32% in low-risk, 55% in moderate, 79% in high, and 89% in very-high risk category over a median of 8.7 (1.7 to 12.8) years (Figure 3C). When assessing MI alone as outcome, the risk was higher in the high- and very high-risk categories (4% in the low-risk, 10% in the moderate, 13% in the high and 12% in the very high-risk category) (C-statistics 0.658 [95% CI: 0.626-0.690]).

Results were consistent after excluding patients with metastatic disease (n = 429): CVD incidence was 7% in low-risk, 22% in moderate-risk, 34% in high-risk, and 47% in the very high-risk category. When excluding those not surviving the first 2 years (n = 633), C-statistics for CVD events were 0.700 (95% CI: 0.673-0.726) and for HF hospitalizations 0.778 (95% CI: 0.737-0.819) (Supplemental Figure 4).

### Performance in patients receiving potentially cardiotoxic treatment

Among patients treated with anthracyclines (n = 255), the most common cancer types were breast (49%) and hematological malignancies (28%) (Supplemental Table 5). In total, 50% (n = 127) were assigned to low-risk, 37% (n = 94) to moderate, and 13% (n = 34) to high/very high risk. CVD incidence during the median 10.6 [2.5-12.9] years follow-up increased with risk category: 4% in low risk, 18% in moderate risk and 24% in high/very high risk (Supplemental Figure 5). The C-statistic was 0.746 (95% CI: 0.676-0.812). For HF hospitalizations alone, the C-statistic was 0.875 (95% CI: 0.813-0.937).

In the group receiving anthracyclines, HER2-targeted therapy, VEGF inhibitors, or myeloma treatment (n = 347), CVD incidence over the median 7.0 [2.1-12.2] years was highest in the highest HFA-ICOS risk categories (3% in low risk, 17% in moderate risk, and 21% in high/very high risk) (Supplemental Figure 6).

### Associations between individual predictors and CVD risk

**Figure 5:**
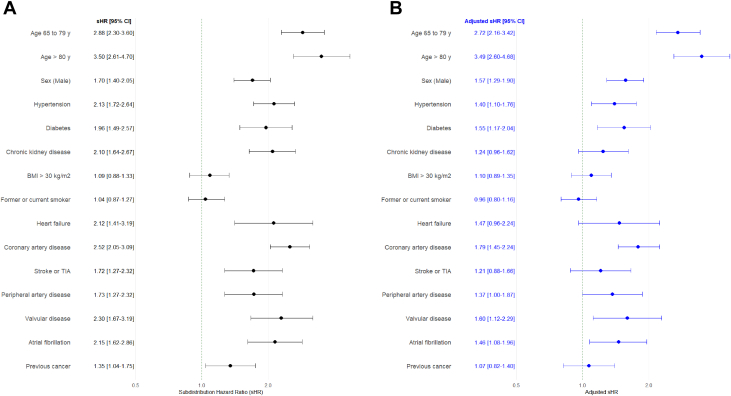
**Predictors of Cardiovascular Events** Forest plot of subdistribution HRs (sHRs) for predictors in the HFA-ICOS tool and cardiovascular event risk. (A) Unjadusted sHRs; (B) adjusted sHR for age and sex. BMI = body mass index; TIA = transient ischemic attack.

Subdistribution HRs for individual predictors in the HFA-ICOS tool are shown in Figure 5. After adjustment for age and sex, diabetes mellitus (sHR: 1.55; 95% CI: 1.17-2.04), hypertension (sHR: 1.40; 95% CI: 1.10-1.76), coronary artery disease (sHR: 1.79; 95% CI: 1.45-2.24), atrial fibrillation (sHR: 1.46; 95% CI: 1.08-1.96), and significant valvular disease (sHR: 1.60; 95% CI: 1.12-2.29) were associated with increased CVD risk. BMI, whether analyzed as a continuous or categorical variable, showed no significant association. Similar trends were observed among anthracycline-treated patients (data not shown).

## Discussion

### Main findings

Our key findings include that: 1) a modified HFA-ICOS tool stratifies CVD outcomes across cancer types; 2) higher-risk categories were associated with more CVD events, HF hospitalizations, and all-cause mortality; 3) the tool shows an overall C-statistic of 0.696; 4) similar risk patterns were found in anthracycline-treated patients; 5) notable heterogeneity existed within low-to-moderate risk groups, which accounted for a high absolute number of events; and 6) key predictors included diabetes, hypertension, coronary artery disease, atrial fibrillation, and valvular disease, after adjusting for age and sex.

### Comparison with other studies assessing performance of the HFA-ICOS tool

We hypothesized that the HFA-ICOS tool could effectively predict CVD risk across diverse cancer types and treatments, which previous studies have not widely evaluated in a large, heterogeneous cancer populations. Higher-risk categories were associated with more events, aligning with previous studies.^7^^,^^8^^,^^17^, ^18^, ^19^ HFA-ICOS also stratified all-cause mortality, reflecting findings from the CARDIOTOX registry.^9^ The tool performed quite well in the long-term perspective. Other studies assessing the performance of the model have been limited by shorter follow-up periods^9^^,^^11^ and smaller sample sizes.^7^^,^^9^, ^10^, ^11^, ^12^^,^^18^, ^19^, ^20^ Furthermore, these studies have focused on specific types of cancers, mainly confined to breast cancer,^7^, ^8^, ^9^, ^10^^,^^17^^,^^18^^,^^20^^,^^21^ melanoma^11^ or hematological cancers,^12^^,^^17^^,^^19^^,^^21^ or specific types of treatment.^9^^,^^10^^,^^19^ The overall C-statistic of 0.696 is comparable to some external validations but lower than others, presumably reflecting differences in population diversities, endpoint definitions, event rates, and follow-up durations.^7^^,^^9^^,^^17^^,^^22^^,^^23^ Unlike other models, HFA-ICOS has not undergone a formal derivation process or rigorous statistical validation in development cohorts, complicating calibration assessments.^4^^,^^24^

The fixed categories used in the HFA-ICOS may not accurately represent individual risks, particularly within the low-to-moderate groups, which comprise 63% of the cohort. Among these, 7% in low-risk and 21% in moderate-risk categories experienced events. Low-risk assignment, comparable to other studies,^7^^,^^9^ is associated with a low incidence of events, which could allow for longer intervals between follow-ups. The low-moderate risk category shows considerable variability with 6% events for patients with 0 points but a 27% event incidence for those with 4 points—similar to the high-risk category (29%) yet assigned to different follow-up recommendations per guidelines.^1^ This variability necessitates more refined risk stratification. Furthermore, patients with established CVD and HF are automatically classified as high- or very high-risk categories, yet these patients also exhibit significant variability in individual CVD risk.^25^

Regression analyses confirmed traditional CVD risk factors (age, sex, hypertension, and CVD comorbidities) as significant predictors. Yet their impact may depend on interactions with other variables, such as age and sex. Notably, sex is not included in the HFA-ICOS tool. The fixed-point value assignment by the HFA-ICOS tool, which does not account for the differential weighting of moderate-risk predictors, has also been noted by other authors.^7^ Incorporation of regression coefficients might better capture the relative contribution of each factor to the overall risk.

Despite limitations, the tool effectively identifies high-risk patients, consistent with previous findings.^7^^,^^9^^,^^10^^,^^12^^,^^17^ Our findings also support observations from other studies showing that the tool identifies low-risk individuals. For example, 90% of low-risk patients receiving anthracyclines remained event-free, indicating potential for reducing unnecessary follow-ups. However, previous studies have also demonstrated that the HFA-ICOS tool may not adequately identify patients at low absolute risk (9, 10, 23). Notably, low sensitivity leads to potentially missing patients at risk, which might limit the practical utility in clinical settings where identifying at-risk individuals is crucial.

### Strengths and limitations

We applied a modified HFA-ICOS tool to a large, diverse cancer cohort in an explanatory manner, extending its use beyond treatment-specific applications. We defined CVD outcomes as a composite of HF hospitalization, MI, stroke, and CVD death. Other studies primarily focus on cancer therapy-related cardiac dysfunction (based on reduction in left ventricular ejection fraction or global longitudinal strain)^10^^,^^11^ or narrower endpoints such as solely clinical or subclinical HF.^9^, ^10^, ^11^ Although pooling HF with atherosclerotic vascular disease combines conditions with different pathophysiology, restricting outcomes to HF may underestimate the overall CVD burden, especially given the diversity of cardiotoxic profiles across therapies.^26^ Even broader outcomes could have been assessed to capture the diverse complications of cancer therapies.^1^^,^^3^ However, the relevance of different CVD endpoints likely depends on the clinical context and intended use of the tool; in decisions involving high-risk HF-inducing therapies, a more targeted HF endpoint may be most useful, although for broader cardio-oncology risk assessment or survivorship planning, a composite outcome may provide a more comprehensive picture of long-term prognosis. The absence of granular baseline data, such as cardiac biomarkers, echocardiographic findings, and detailed cancer therapy dosing, may limit the accuracy of risk classification, especially in the low- and moderate-risk groups, where such information could conceal clinically relevant variation in underlying CVD risk. However, this reflects a common clinical reality where these data often are not readily available and the reasonable performance without access to advanced diagnostic data suggests feasibility in settings with limited clinical information. Focusing on clinical endpoints provides valuable insights for patient management. That said, there are tradeoffs between enhancing diagnostic sensitivity, such as including subclinical outcomes, and the potential decrease in specificity, which could lead to an increased burden of follow-up testing without necessarily improving outcomes.^27^ Our study was limited by the lack of information on follow-up CVD medications and cancer drugs administered orally. Data on ethnicity were not available; however, the study population is assumed to be predominantly Northern European/Caucasian, which may limit generalizability to more diverse populations. Finally, the study’s applicability may be constrained by advancements in cancer treatments since HUNT3.

### Clinical implications

As survival in cancer care improves, effective CVD risk stratification is increasingly needed. The HFA-ICOS tool, available as a user-friendly app, offers a structured framework based on traditional CVD risk factors and well-known risk patterns, although acknowledging additional risk from cancer and its treatment.^1^^,^^3^^,^^6^^,^^22^ In our exploratory application to a broader cancer population, the tool effectively identified high-risk patients but showed variability within the low-moderate risk group, where improved stratification would be most valuable. Despite moderate sensitivity and specificity, its high negative predictive value suggests clinical utility by providing reassurance and avoiding unnecessary follow-up. Given the limited availability and validation of alternative CVD risk tools in cancer patients,^1^^,^^22^^,^^28^, ^29^, ^30^, ^31^, ^32^ HFA-ICOS remains feasible option, although future studies should explore integration of biomarkers, imaging, and treatment-related variables to enhance discrimination, particularly in lower-risk groups. Although no single tool is likely to capture all complexities of CVD outcomes in cancer patients, a unified continuous basis score, with “add-on” of cancer-specific factors could simplify clinical use and reduce the need for multiple tools or proformas.

## Conclusions

A modified HFA-ICOS tool stratifies future CVD risk on a population level across a broad range of cancer types. These results may be useful in guiding follow-up frequency during and after cancer therapy and optimizing prevention. However, achieving more individualized risk assessment, especially within the low-moderate-risk category, requires tailoring and refining existing CVD risk prediction models.

Perspectives **COMPETENCY IN PATIENT CARE:** A modified HFA-ICOS tool without cardiac biomarkers and imaging data identifies broad CVD risk categories in cancer patients but shows more modest discriminatory ability within the large low-to-moderate risk group, where the majority of events occur, highlighting the need for improved risk stratification and tailored monitoring in this population.

**TRANSLATIONAL OUTLOOK:** Future research should focus on refining risk prediction in the low-to-moderate risk group by integrating biomarkers, imaging, and cancer treatment details.

## Funding support and author disclosures

The authors conducted the work as part of their regular duties at their affiliated institutions. Registry linkage was funded by Clinic of Cardiology and Clinic of Medicine, St. Olavs Hospital. Dr Omland has received speaker or consulting honoraria from 10.13039/100016545Roche Diagnostics, 10.13039/100001316Abbott Laboratories, SpinChip and CardiNor, and research support from 10.13039/100000046Abbott, ChromaDex, 10.13039/100004336Novartis, and 10.13039/100004337Roche. All other authors have reported that they have no relationships relevant to the contents of this paper to disclose.

## References

[1] Lyon A.R., López-Fernández T., Couch L.S. (2022). 2022 ESC guidelines on cardio-oncology developed in collaboration with the European hematology association (EHA), the European society for therapeutic radiology and oncology (ESTRO) and the International cardio-oncology society (IC-OS): developed by the task force on cardio-oncology of the European society of cardiology (ESC). Eur Heart J.

[2] Raisi-Estabragh Z., Murphy Alexandra C., Ramalingam S. (2024). Cardiovascular considerations before cancer therapy. JACC: CardioOncology.

[3] Lyon A.R., Dent S., Stanway S. (2020). Baseline cardiovascular risk assessment in cancer patients scheduled to receive cardiotoxic cancer therapies: a position statement and new risk assessment tools from the cardio-oncology study group of the heart failure association of the European society of cardiology in collaboration with the International cardio-oncology society. Eur J Heart Fail.

[4] Rossello X., Dorresteijn J.A., Janssen A. (2019). Risk prediction tools in cardiovascular disease prevention: a report from the ESC prevention of CVD programme led by the European association of preventive cardiology (EAPC) in collaboration with the Acute cardiovascular care association (ACCA) and the Association of cardiovascular nursing and allied professions (ACNAP). Eur Heart J Acute Cardiovasc Care.

[5] Moons K.G., Royston P., Vergouwe Y., Grobbee D.E., Altman D.G. (2009). Prognosis and prognostic research: what, why, and how?. BMJ.

[6] Okwuosa T.M., Keramida K., Filippatos G., Yancy C.W. (2020). Cancer therapy and the heart; the necessity to calibrate risk. Eur J Heart Fail.

[7] Cronin M., Crowley A., Davey M.G. (2023). Heart failure association-international cardio-oncology society risk score validation in HER2-Positive breast cancer. J Clin Med.

[8] Suntheralingam S., Fan C.P.S., Calvillo-Argüelles O., Abdel-Qadir H., Amir E., Thavendiranathan P. (2022). Evaluation of risk prediction models to identify cancer therapeutics related cardiac dysfunction in women with HER2+ breast cancer. J Clin Med.

[9] Rivero-Santana B., Saldaña-García J., Caro-Codón J. (2025). Anthracycline-induced cardiovascular toxicity: validation of the heart failure association and international cardio-oncology society risk score. Eur Heart J.

[10] Battisti N.M.L., Andres M.S., Lee K.A. (2021). Incidence of cardiotoxicity and validation of the heart failure association-International cardio-oncology society risk stratification tool in patients treated with trastuzumab for HER2-positive early breast cancer. Breast Cancer Res Treat.

[11] Glen C., Adam S., McDowell K. (2023). Cardiotoxicity of BRAF/MEK inhibitors: a longitudinal study incorporating contemporary definitions and risk scores. JACC: CardioOncology.

[12] Di Lisi D., Madaudo C., Alagna G. (2022). The new HFA/ICOS risk assessment tool to identify patients with chronic myeloid leukaemia at high risk of cardiotoxicity. ESC Heart Fail.

[13] Krokstad S., Langhammer A., Hveem K. (2013). Cohort profile: the HUNT study, Norway. Int J Epidemiol.

[14] Cancer Registry of Norway. Cancer in Norway. 2022. http://www.fhi.no/globalassets/cancer-in-norway/2022/cin_report-2022.pdf.

[15] Enerly E., Holmstrøm L., Skog A. (2021). INSPIRE: a new opportunity for cancer pharmacoepidemiology research. Norsk Epidemiologi.

[16] Knopfholz J., Disserol C.C., Pierin A.J. (2014). Validation of the friedewald formula in patients with metabolic syndrome. Cholesterol.

[17] Soh C.H., Marwick T.H. (2024). Comparison of heart failure risk assessment tools among cancer survivors. Cardiooncology.

[18] Tini G., Cuomo A., Battistoni A. (2022). Baseline cardio-oncologic risk assessment in breast cancer women and occurrence of cardiovascular events: the HFA/ICOS risk tool in real-world practice. Int J Cardiol.

[19] Fernando F., Andres M.S., Claudiani S. (2024). Cardiovascular events in CML patients treated with nilotinib: validation of the HFA-ICOS baseline risk score. Cardiooncology.

[20] Di Lisi D., Madaudo C., Faro D.C. (2024). The added value of the HFA/ICOS score in the prediction of chemotherapy-related cardiac dysfunction in breast cancer. J Cardiovasc Med (Hagerstown).

[21] Shibata T., Nohara S., Morikawa N. (2023). Cardiovascular adverse events and prognosis in patients with haematologic malignancies and breast cancer receiving anticancer agents: kurume-CREO registry insights. Eur J Prev Cardiol.

[22] McCracken C., Condurache D.-G., Szabo L. (2024). Predictive performance of cardiovascular risk scores in cancer survivors from the UK biobank. JACC CardioOncology.

[23] Cook N.R. (2007). Use and misuse of the receiver operating characteristic curve in risk prediction. Circulation.

[24] Van Calster B., McLernon D.J., van Smeden M., Wynants L., Steyerberg E.W. (2019). Calibration: the achilles heel of predictive analytics. BMC Med.

[25] Kaasenbrood L., Boekholdt S.M., van der Graaf Y. (2016). Distribution of estimated 10-Year risk of recurrent vascular events and residual risk in a secondary prevention population. Circulation.

[26] Rye C.S., Ofstad A.P., Åsvold B.O., Romundstad P.R., Horn J., Dalen H. (2024). The influence of diagnostic subgroups, patient- and hospital characteristics for the validity of cardiovascular diagnoses–data from a Norwegian hospital trust. PLoS One.

[27] Yeh E.T., Vejpongsa P. (2015). Subclinical cardiotoxicity associated with cancer therapy: early detection and future directions. J Am Coll Cardiol.

[28] Kim D.Y., Park M.S., Youn J.C. (2021). Development and validation of a risk score model for predicting the cardiovascular outcomes after breast cancer therapy: the CHEMO-RADIAT score. J Am Heart Association.

[29] Abdel-Qadir H., Thavendiranathan P., Austin P.C. (2019). Development and validation of a multivariable prediction model for major adverse cardiovascular events after early stage breast cancer: a population-based cohort study. Eur Heart J.

[30] Ezaz G., Long J.B., Gross C.P., Chen J. (2014). Risk prediction model for heart failure and cardiomyopathy after adjuvant trastuzumab therapy for breast cancer. J Am Heart Assoc.

[31] Liu X., Tao L., Wang M., Li H., Xu W. (2022). ABSDELL model: development and internal validation of a risk prediction model of LVEF decline in breast cancer patients treated with trastuzumab. Clin Breast Cancer.

[32] Kaboré E.G., Macdonald C., Kaboré A. (2023). Risk prediction models for cardiotoxicity of chemotherapy among patients with breast cancer: a systematic review. JAMA Network Open.

