# Supplementary material for: Prognostic Performance of a Modified HFA-ICOS Tool for Prediction of Cardiovascular Complications in Cancer Patients
Source: JACC Adv. 2025 Nov 14;4(12):102344. doi: 10.1016/j.jacadv.2025.102344 (PMC12663498; doi:10.1016/j.jacadv.2025.102344)
Supplement: Supplemental Material [file mmc1.pdf]

Supplementary tables and figures

<u>Content</u>

- Figure S1. Study design
- Table S1. Diagnostic codes from the hospital registry used to define cardiovascular comorbidities and outcomes
- Supplementary Methods
- Table S2. Assignment of risk levels in by the HFA-ICOS risk stratification tool and availability of predictors.
- Table S3. Patient, disease and tumor characteristics by cancer subtype
- Table S4. Proportions of participants receiving treatment according to the 7 classes of potentially cardiotoxic treatments
- Table S5. Characteristics for patients receiving anthracyclines, HER2 targeted therapy, intravenous VEGF inhibitors and multiple myeloma treatment
- Table S6. Number of CVD events per HFA-ICOS risk category
- Figure S2. Interval incidence per 6 months for cardiovascular events and non-cardiovascular deaths for the overall population.
- Figure S3. Cumulative incidence plot for CV complications adjusted for competing risk of non-CVD death during follow-up
- Figure S4**.** Survival curves for cardiovascular events (A), and heart failure hospitalization (B) among patients at low, moderate, high and very high risk according to the HFA-ICOS risk stratification tool for patients surviving the first two years (n=1657).
- Figure S5. Kaplan Meier survival curves for patients receiving anthracyclines
- Figure S6. Kaplan Meier survival curves for patients receiving anthracyclines, HER2-targeted therapy, VEGF inhibitors or myeloma treatment

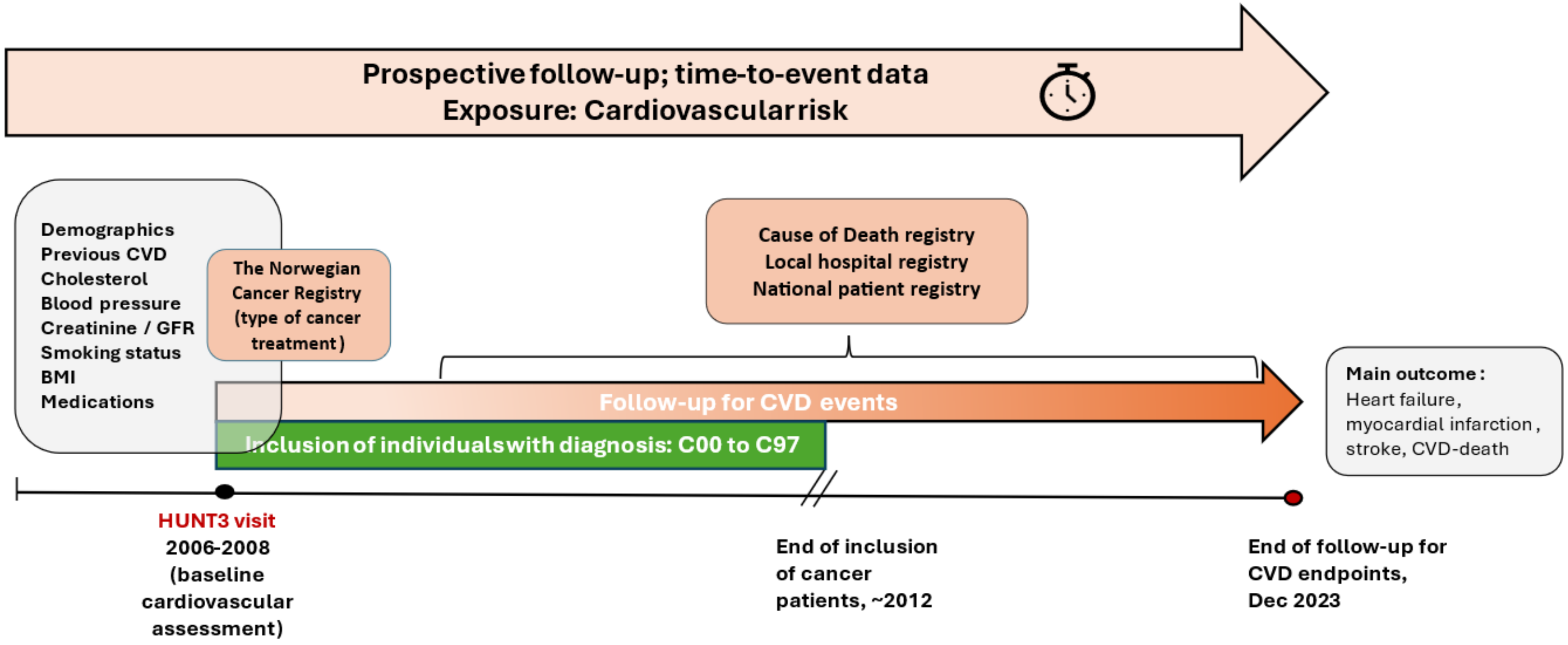

Figure S1. Study design

Table S1. Definition of the study population, baseline variables and cardiovascular endpoints			
	**Variable**	**Definition**	**Source**
**Definition of study population**			
	Cancer diagnosis between HUNT3 and 2012	Any malignancy, including leukemia and lymphoma, including ICD-9-codes: 140-209 and ICD-10-codes: C00-C96. D00-D49 (in situ and benign neoplasms were excluded).	NCR
**Definition of baseline variables**			
	Chemotherapy	Yes / no. Reported by clinicians.	NCR
	Anthracyclines	ATC-code: L01DB	NCR (INSPIRE-project*)
	Anti HER2 treatment	ATC-code: L01FD	NCR (INSPIRE-project*)
	Radiotherapy	Yes / no. Reported by clinicians.	NCR
	Left breast radiotherapy	Region reported by clinicians.	NCR
	Mediastinal radiotherapy	Region reported by clinicians.	NCR
	Surgery	Yes / no. Reported by clinician.	NCR
	Smoking	Self-reported. «Do you smoke?» Options: No, never. No, quitted. Yes, occasionally. Yes, daily.	HUNT3
	Diabetes	Self-reported diabetes or diabetes diagnosis in hospital medical records	Local hospital registry and HUNT3
	Body Mass Index (kg/m^2)	Weight and height (kg/m^2)	HUNT3
	Systolic blood pressure (mmHg)	Measured manually at the HUNT3 visit twice with one minute interval by specially trained nurses. Measured on the right arm with cuffs adjusted according to the arm circumference, and after the participant had been sitting relaxed for five minutes.	HUNT3
	Diastolic blood pressure (mmHg)	Same as systolic blood pressure.	HUNT3
	Antihypertensiva	Self-reported. «Do you take or have you taken medication for high blood pressure?” Yes/no.	HUNT3

	Hypertension	Use of antihypertensive drugs or baseline blood pressure > 140/90 mmHg	HUNT3
	Chronic kidney disease	Estimated glomerular filtration rate (eGFR) < 60 ml/min/1.73 m^2 or CKD diagnosis in hospital medical records	
	Total cholesterol (mmol/L)	Venous blood samples	HUNT3
	HDL cholesterol (mmol/L)	Venous blood samples	HUNT3
	LDL cholesterol (mmol/L)[c]	Calculated using the Friedewald formula.	HUNT3
	Coronary artery disease	**ICD-9*:** 410, 411, 412, 413, 414, 429.2, 429.7, V45.81, V45.82<br>**ICD-10*:** I20, I21, I22, I23, I24, I25, Z95.1, Z95.5, Z98.61	Local hospital registry and HUNT3
	Cerebrovascular	**ICD-9:** 362.34, 430, 431, 432, 433, 434, 435, 436, 437, 438<br>**ICD-10:** I61, I62, I63, I65, I66, I67, I68, I69, G45, G46, H34.0	Local hospital registry and HUNT3
	Peripheral artery disease	**ICD-9:** 440, 441, 443.89, 443.9, 444, 447.1, 557.1, 557.9, V43.4<br>**ICD-10:** I70, I71, I73.8, I73.9, I74, I77.1, I79.0, K55.1, K55.8, K55.9, Z95.82, Z98.62	Local hospital registry and HUNT3
	Heart failure	**ICD-9:** 398.91, 402.01, 402.11, 402.91, 404.01, 404.03, 404.11, 404.13, 404.91, 404.93, 425.4–425.9, 428<br>**ICD-10:** I11.0, I13.0, I13.2, I42.0, I42.6-I42.9, I43, I50	Local hospital registry and HUNT3
	Atrial fibrillation	ICD-9: 427.3<br>ICD-10: I48	Local hospital registry and HUNT3
**Definition of cardiovascular endpoints**			
	Myocardial infarction	ICD-10 codes I21, I22 and I24 (main or secondary diagnosis) (2).	Local hospital registry

Stroke	ICD-10 codes I61, I63 and I64 (main diagnosis) (3).	Local hospital registry
Cardiovascular death	ICD-10 I00-I99 registered as the primary cause of death	Local hospital registry
Non-cardiovascular death	All other causes of death except cardiovascular.	Local hospital registry

Abbreviations: ICD; international classification of diseases, ATC; anatomic therapeutic classification, NCR; The Norwegian Cancer Registry. *= The INSPIRE (INcreaSe PharmaceutIcal REporting) project.

Supplementary methods

Assignment of risk levels according to the HFA-ICOS tool

All 2,290 patients were assigned to one of four cardiovascular (CV) risk categories – low, moderate, high, or very high – according to the HFA-ICOS risk stratification tool. This tool provides treatment-specific algorithms for seven classes of cancer therapies with known cardiotoxic potential: anthracyclines, HER2-targeted therapies, VEGF inhibitors, BCR-ABL inhibitors, multiple myeloma treatments, and RAF/MEK inhibitors. However, several of these therapies (e.g., RAF/MEK and BCR-ABL inhibitors) were not yet approved or in routine clinical use during the study period (2006–2008), and information on oral drugs was not available. In addition, diagnostic data such as electrocardiograms (ECG), echocardiography, and cardiac biomarkers (e.g., troponin, NT-proBNP) were not available, nor were certain clinical factors (e.g., pulmonary hypertension, ankle–brachial index, proteinuria, venous thrombosis, or use of dexamethasone).

Given these limitations, and to reflect the ESC guideline recommendation of pre-treatment CV risk stratification in all cancer patients regardless of treatment type, we applied a pragmatic and harmonized version of the HFA-ICOS scoring system to the entire cohort. The risk classification was based on the available predictors (e.g., age, hypertension, diabetes, chronic kidney disease, smoking, obesity, prior CVD, and previous cardiotoxic treatment), which largely reflects the predictors often available in routine clinical care. For overlapping predictors, point allocation was generally consistent across the treatment-specific algorithms, with only minor differences.

Patients were categorized as follows:

- **Low risk**: No risk factors or one medium-level factor (1 point)
- **Moderate risk**: 2–4 medium-level risk points
- **High risk**: ≥5 medium-level points or any high-risk factor
- **Very high risk**: Presence of any very-high-risk factor

This approach allowed for uniform risk stratification regardless of type of treatment and the evolving therapy landscapes. In secondary analyses, we stratified patients according to the actual cancer therapy they received, using data from the Norwegian Cancer Registry's INSPIRE project, which captures intravenously administered systemic cancer therapies given in hospital settings. Further details and the complete point-based stratification are provided in Supplementary Table S2.

Table S2. Assignment of risk levels based on HFA-ICOS for selected cancer therapies and for the overall population

	Anthracyclines	VEGF inhibitors	HER2 targeted therapy	Multiple myeloma treatment	Pragmatic assignment for the overall population[d]
**Previous CVD**					
Heart failure or cardiomyopathy	Very high	Very high	Very high	Very high	Very high
Severe valvular heart disease	High	-	High		Not available[e]
Myocardial infarction or previous coronary revascularization (PCI or CABG)	High	Very high	High		High
Arterial vascular disease (PAD, TIA, stroke)		Very high		Very high	High
Stable angina	High	Very high	High		High
Arrythmia[a]		Medium 2p	Medium 2p	Medium 2p	Medium 2p
**Cardiac imaging**					
Baseline LVEF < 50%	Not available	Not available	Not available	Not available	Not available
Borderline LVEF 50–54%	Not available	Not available	Not available	Not available	Not available
LV hypertrophy				Not available	Not available
**Cardiac biomarkers**					
Elevated baseline troponin	Not available	Not available	Not available	Not available	Not available
Elevated baseline NT-proBNP	Not available	Not available	Not available	Not available	Not available
**Demographic and cardiovascular risk factors**					
Age ≥ 80 years	High	High[b]	High	High	High
Age 65–79 years	Medium 2p	Medium 1p	Medium 2p	Medium 1p	Medium 2p

Hypertension	Medium 1p	High	Medium 1p	Medium 1p	Medium 1p
Diabetes mellitus	Medium 1p	Medium 1p	Medium 1p	Medium 1p	Medium 1p
Hyperlipidemia[C]		Medium 1p			Medium 1p
Chronic kidney disease	Medium 1p	Medium 1p	Medium 1p	Medium 1p	Medium 1p
Current smoker	Medium 1p	Medium 1p	Medium 1p	Medium 1p	Medium 1p
Obesity (BMI >30 kg/m^2)	Medium 1p	Medium 1p	Medium 1p	Medium 1p	Medium 1p
**Previous cardiotoxic cancer treatment**					
Previous anthracycline exposure	High	High	Medium 2p	High	High
Prior RT to left chest or mediastinum	High	Medium 1p	Medium 2p	Medium 1p	Medium 1p
Previous non-anthracycline-based chemotherapy	Medium 1 p	-			Medium 1p

Abbreviations: *CABG, coronary artery bypass grafting; CV, cardiovascular; CVD, cardiovascular disease; HER2, human epidermal growth factor receptor 2; LVEF, left ventricular ejection fraction; NT-proBNP, N-terminal pro–B-type natriuretic peptide; PAD, peripheral artery disease; PCI, percutaneous coronary intervention; p, points; RT, radiotherapy; TIA, transient ischemic attack; VEGF, vascular endothelial growth factor.*

a Atrial fibrillation, atrial flutter, ventricular tachycardia, or ventricular fibrillation.

b In the original HFA-ICOS scheme, age ≥75 years was classified as high risk for VEGF inhibitors.

c Non–high-density lipoprotein cholesterol >3.8 mmol/L.

d Pragmatic risk assignment reflects a harmonized scoring approach applied to the entire cohort, regardless of actual cancer therapy received. When risk weights differed between treatment-specific HFA-ICOS algorithms (e.g., for age or hypertension), the most frequently assigned risk level was used to ensure consistency.

e Severity of valvular heart disease was not available.

Table S3. Patient, disease and tumor characteristics by cancer subtype								
	GI cancer (n=529)	Lung cancer (n=207)	Melanoma (n=272)	Breast cancer (n=273)	Female genital cancer (n=133)	Prostate cancer (n=425)	Hematological cancer (n=179)	Other solid tumors[b] (n=272)
Age, mean (SD)	69.2 (11)	69.3 (9.4)	65.3 (14)	59.1 (12.3)	62.2 (13.5)	65.9 (11.2)	66.5 (13.2)	65.1 (13.9)
Age >75 years	196 (37%)	72 (35%)	87 (32%)	38 (14%)	24 (18%)	93 (22%)	57 (32%)	78 (29%)
Sex, female	275 (52%)	119 (57%)	137 (50%)	273 (100%)	133 (100%)	0 (0%)	103 (57%)	135 (50%)
Diabetes mellitus	52 (10%)	15 (7%)	28 (10%)	19 (7%)	5 (4%)	34 (8%)	13 (7%)	20 (7%)
Coronary artery disease	99 (19%)	47 (23%)	41 (15%)	16 (6%)	7 (5%)	77 (18%)	28 (17%)	41 (15%)
Cerebrovascular disease	40 (8%)	24 (12%)	15 (6%)	11 (4%)	7 (5%)	35 (8%)	12 (7%)	19 (7%)
Peripheral arterial disease	39 (7%)	30 (15%)	15 (6%)	6 (2%)	4 (3%)	30 (7%)	7 (4%)	15 (6%)
Heart failure	26 (5%)	7 (3%)	8 (3%)	6 (2%)	3 (2%)	10 (2%)	7 (4%)	1 (0%)
Atrial fibrillation	57 (11%)	20 (10%)	23 (8%)	7 (3%)	7 (5%)	43 (10%)	17 (10%)	12 (4%)
Chronic kidney disease	69 (13%)	27 (13%)	39 (14%)	14 (5%)	15 (11%)	32 (8%)	22 (12%)	28 (10%)
SEER staging[a]								
1 (Localized)	94 (18%)	36 (17%)	221 (81%)	173 (63%)	65 (49%)	195 (46%)	21 (11%)	150 (55%)
2 (Regional)	245 (46%)	68 (33%)	6 (2%)	82 (30%)	21 (16%)	99 (23%)	15 (8%)	38 (14%)
3 (Distant)	156 (30%)	91 (44%)	3 (1%)	3 (1%)	41 (31%)	47 (11%)	54 (30%)	34 (13%)
Unstaged	34 (6%)	12 (6%)	42 (15%)	15 (5%)	6 (5%)	84 (20%)	89 (50%)	50 (18%)
Chemotherapy	172 (33%)	110 (53%)	39 (14%)	141 (52%)	75 (56%)	74 (17%)	121 (68%)	64 (24%)
Radiotherapy	86 (16%)	94 (45%)	34 (13%)	201 (74%)	41 (31%)	163 (38%)	40 (22%)	102 (38%)
Surgery	385 (73%)	65 (31%)	249 (92%)	269 (99%)	114 (86%)	204 (48%)	25 (14%)	209 (77%)
Smoking	122 (24%)	105 (54%)	46 (18%)	84 (32%)	26 (21%)	84 (20%)	34 (20%)	55 (22%)
Body Mass Index (kg/m^2)	27.4 (4.2)	26.5 (4.3)	27.5 (3.7)	27.9 (4.9)	28.5 (5.3)	27.3 (3.3)	27.4 (4.1)	27.8 (4.3)

Systolic blood pressure (mmHg)	139 (20)	137 (20)	136 (19)	133 (20)	133 (21)	140 (20)	136 (19)	137 (21)
Antihypertensiva	230 (44%)	82 (40%)	119 (44%)	74 (27%)	42 (32%)	164 (39%)	62 (35%)	109 (40%)
Total cholesterol (mmol/L)	5.6 (1.2)	5.6 (1.1)	5.6 (1.2)	5.8 (1.0)	5.8 (1.0)	5.5 (1.1)	5.3 (1.2)	5.6 (1.1)
HDL cholesterol (mmol/L)	1.3 (0.4)	1.4 (0.4)	1.4 (0.4)	1.5 (0.4)	1.4 (0.4)	1.2 (0.3)	1.3 (0.3)	1.4 (0.4)

[a]Stage of cancer disease according to SEER (U.S. National Cancer Institute's Surveillance, Epidemiology and End Results) Program. [b]Cancer of lip, oral cavity and pharynx, bone cancer, mesothelial or soft tissue cancer, central nervous system cancer, testis cancer, or endocrine cancer. Abbreviations: GI, gastrointestinal; HDL, high-density lipoprotein.

Table S4. Types of intravenous treatments by the 7 classes of potentially cardiotoxic medications covered by the HFA-ICOS tool (4, 5)				
	**ATC codes**	**Name**	**Administration**	**n**
Anthracyclines				255
	L01DB01	Doxorubicin	Iv	110
	L01DB02	Daunorubicin	Iv	8
	L01DB03	Epirubicin	Iv	140
	L01DB06	Idarubicin	Iv	1
	L01DB07	Mitoxantrone	Iv	1
HER 2 targeted therapy				29
	L01FD01	Trastuzumab	Iv	29
	L01FD02	Pertuzumab	Iv	1
VEGF inhibitors				52
	L01FG01	Bevacizumab	Iv	52
Multiple myeloma treatment		Data only partly available*	Iv/po	41
BRC-ABL tyrosin kinase inhibitors		Data not available	po	-
RAF inhibitors		Data not available	po	-
MEK inhibitors		Data not available	po	-

Abbreviations: ATC, Anatomical Therapeutic Chemical classification; HER2, Human Epidermal Growth Factor Receptor 2; Iv, intravenously; PO, per os; VEGF, vascular endothelial growth factor. Comments: BCR-ABL (Breakpoint Cluster Region-Abelson) tyrosine kinase inhibitors are mainly administered po and data were not available. RAF and MEK inhibitors were approved mainly after the conduction of this study. *We assumed that all patients diagnosed with multiple myeloma received some form of treatment.

Table S5. Characteristics for patients receiving anthracyclines, HER2 targeted therapy, intravenous VEGF inhibitors and myeloma treatment				
	**Anthracyclines (n=255)**	**Trastuzumab / pertuzumab (n=29)**	**VEGF inhibitors (n=52)**	**Myeloma (n=41)**
Age, mean (SD)	57.5 (12.5)	52.3 (10.2)	61.3 (9.3)	69.2 (10.3)
Age >75 y	25 (10%)	1 (3%)	0 (0%)	15 (37%)
Sex, female	181 (71%)	29 (100%)	18 (35%)	28 (68%)
**Cancer type**				
Gastrointestinal cancer	13 (5%)	0 (%)	42 (81%)	0 (%)
Lung cancer	7 (3%)	0 (%)	1 (2%)	0 (%)
Breast cancer	125 (49%)	28 (97%)	1 (2%)	0 (%)
Female genital cancer	12 (5%)	0 (%)	1 (2%)	0 (%)
Prostate cancer	0 (%)	0 (%)	0 (0%)	0 (%)
Hematological cancer	72 (28%)	0 (%)	2 (4%)	41 (100%)
Other solid tumors	26 (10%)	1 (3%)	5 (10%)	0 (%)
Metastatic disease	57 (22%)	1 (3%)	26 (50%)	-
**Cardiovascular disease**				
Coronary artery disease	11 (4%)	0 (0%)	5 (10%)	9 (22%)
Peripheral artery disease	6 (2%)	0 (0%)	1 (2%)	3 (7%)
Heart failure	3 (1%)	1 (3%)	0 (0%)	4 (10%)
Atrial fibrillation	6 (2%)	0 (0%)	2 (4%)	7 (17%)
Valvular disease	1 (0%)	0 (0%)	1 (2%)	2 (5%)
Previous stroke or TIA	8 (3%)	0 (0%)	1 (2%)	4 (10%)
**Cardiovascular risk factors**				
Smoking	67 (27%)	9 (31%)	12 (23%)	8 (20%)
Antihypertensives	56 (22%)	2 (7%)	14 (27%)	17 (42%)
Systolic blood pressure	133 (20)	129 (18)	130 (18)	139 (18)
Chronic kidney disease	14 (5%)	0 (0%)	1 (2%)	7 (17%)
Diabetes mellitus	8 (3%)	1 (3%)	2 (4%)	4 (10%)
Cholesterol	5.6 (1.1)	5.6 (0.8)	5.7 (1.1)	5.1 (1.1)
HDL-C	1.4 (0.3)	1.5 (0.4)	1.3 (0.3)	1.2 (0.3)
LDL-C	3.9 (1.0)	3.8 (0.7)	4.1 (1.0)	3.5 (1.0)

BMI	28 (5)	28 (6)	27 (4)	28 (4)
Obesity (BMI > 30)	69 (27%)	7 (24%)	13 (25%)	12 (29%)
**Other cancer therapies**				
Surgery	186 (73%)	28 (97%)	38 (73%)	1 (2%)
Hormonal therapy	66 (26%)	15 (52%)	2 (4%)	0 (0%)
Radiotherapy	152 (60%)	23 (79%)	20 (38%)	12 (29%)

Abbreviations: HDL-C; high-density lipoprotein cholesterol, LDL-C; low-density lipoprotein cholesterol, BMI; body mass index, VEGF; vascular endothelial growth factor.

Table S6. First cardiovascular events per HFA ICOS risk category

	Total (n= 2290)	Low (n=631)	Moderate (n=806)	High (n=785)	Very high (n=68)
No event during follow-up	827 (36%)	405 (64%)	295 (37%)	121 (15%)	6 (9%)
Myocardial infarction	161 (7%)	19 (3%)	60 (7%)	79 (10%)	3 (4%)
Stroke	130 (6%)	16 (3%)	56 (7%)	57 (7%)	1 (2%)
HF hospitalization	87 (4%)	1 (0%)	27 (3%)	46 (6%)	13 (19%)
CVD-death	82 (4%)	6 (1%)	21 (3%)	47 (6%)	8 (12%)
Non-CVD death	1003 (44%)	184 (29%)	347 (43%)	435 (55%)	37 (54%)

Abbreviations: CVD; cardiovascular disease, HF; heart failure

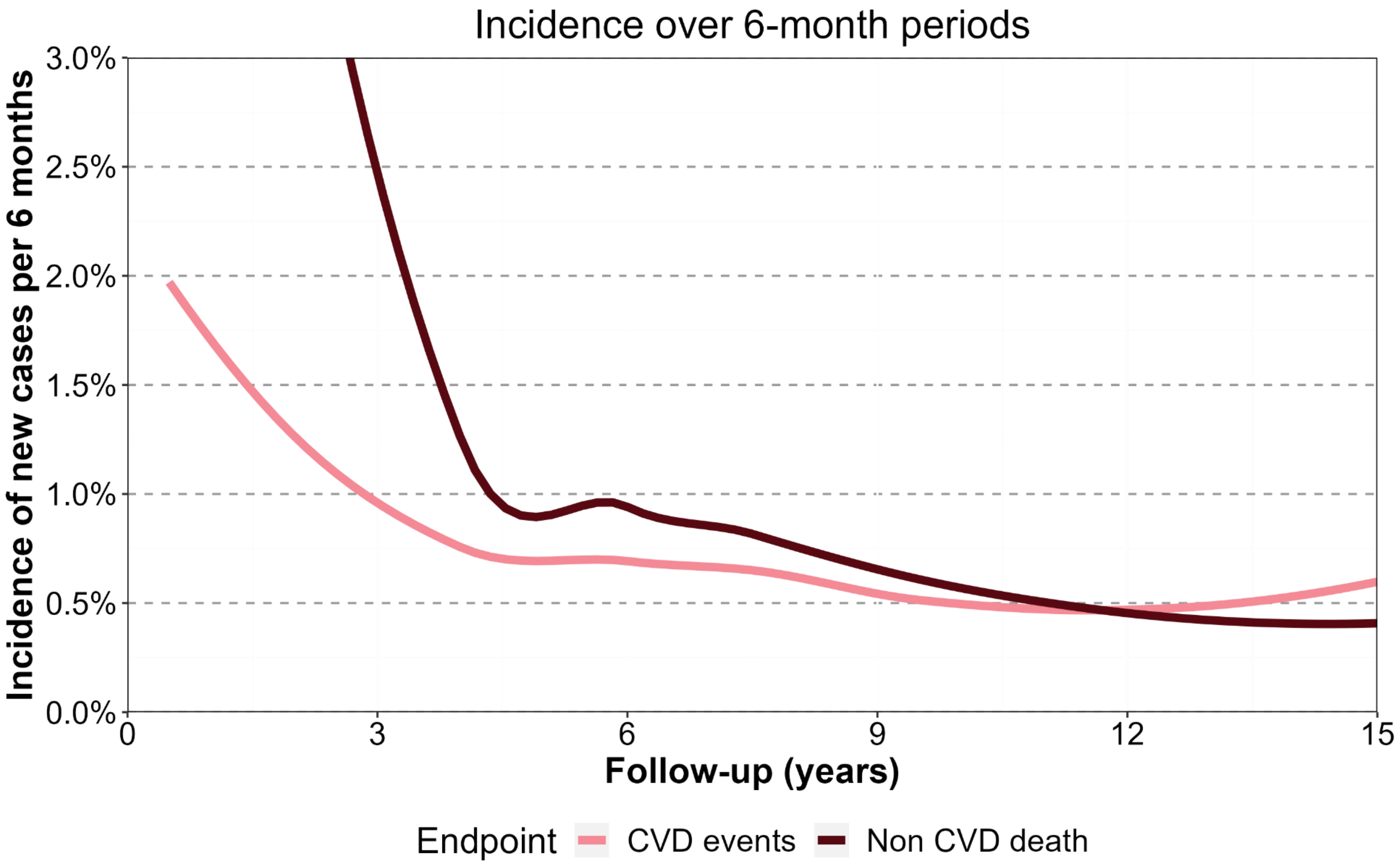

Figure S2. Interval incidence per 6 months for cardiovascular events and non-cardiovascular deaths for the overall population. *Abbreviations: CVD; cardiovascular disease*

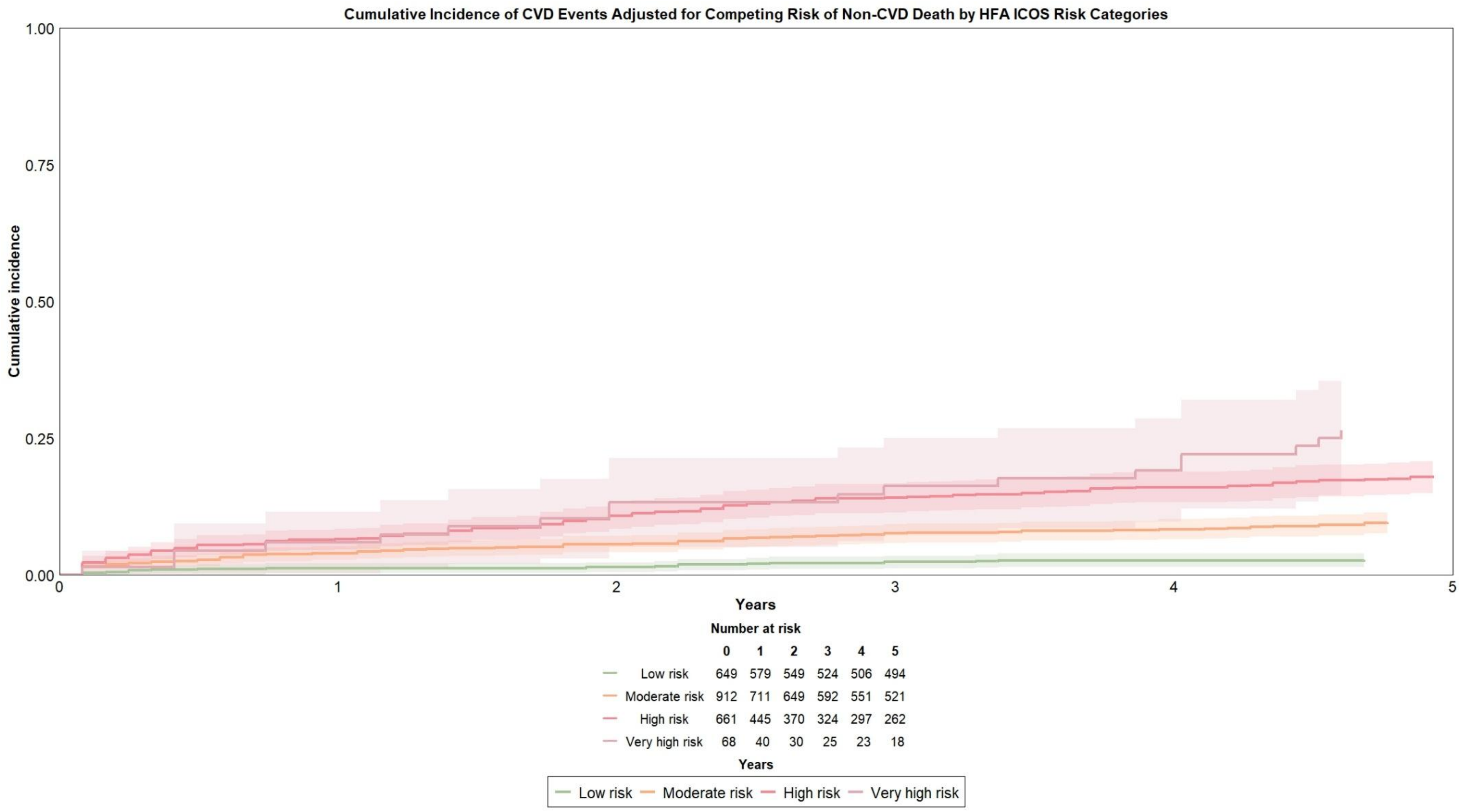

Figure S3. Cumulative incidence of CVD events adjusted for competing risk of non-CVD death by HFA-ICOS risk categories.

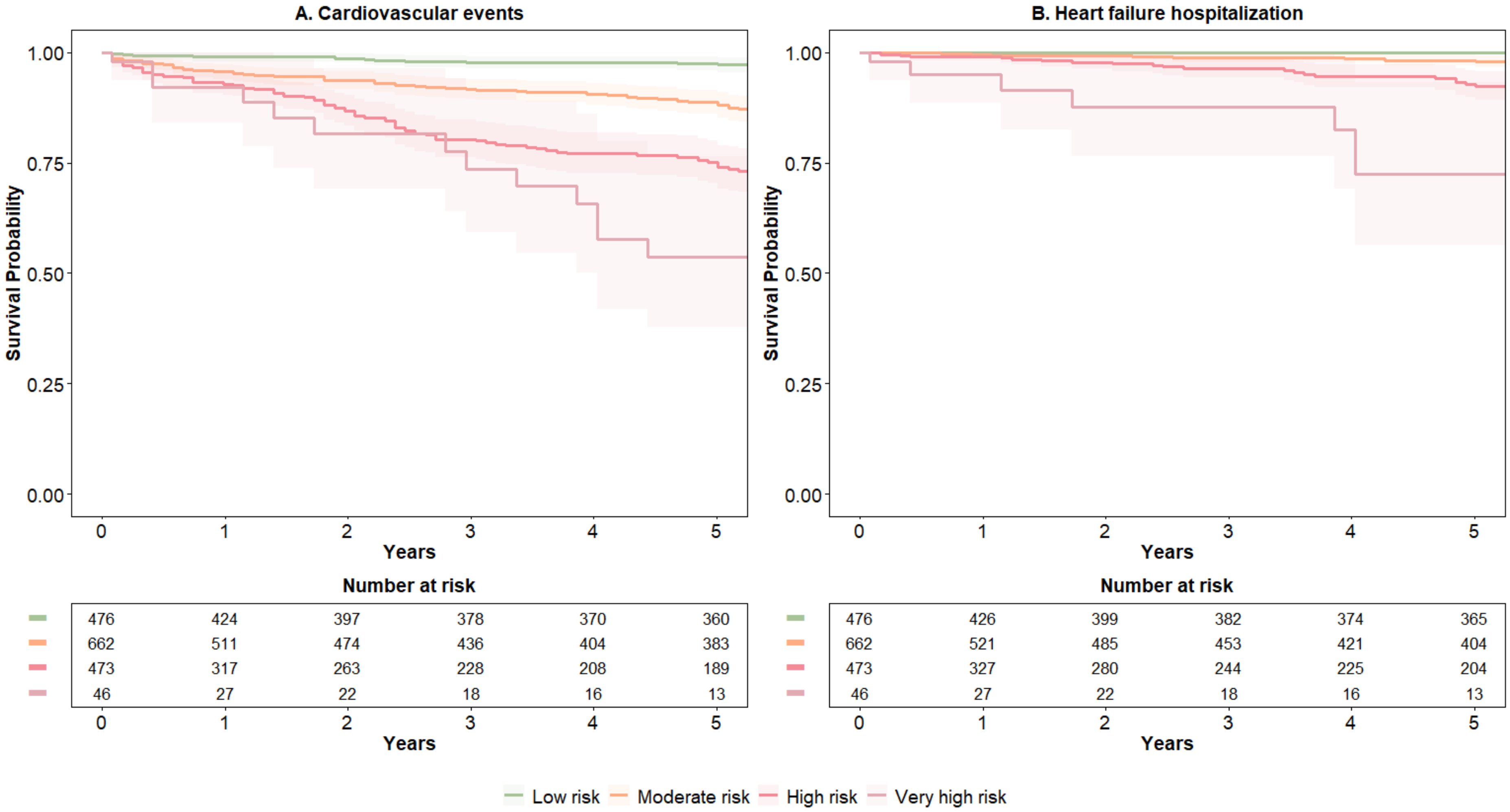

Figure S4. Survival curves for cardiovascular events (A), and heart failure hospitalization (B) among patients at low, moderate, high and very high risk according to the HFA-ICOS risk stratification tool for patients surviving the first two years (n=1657).

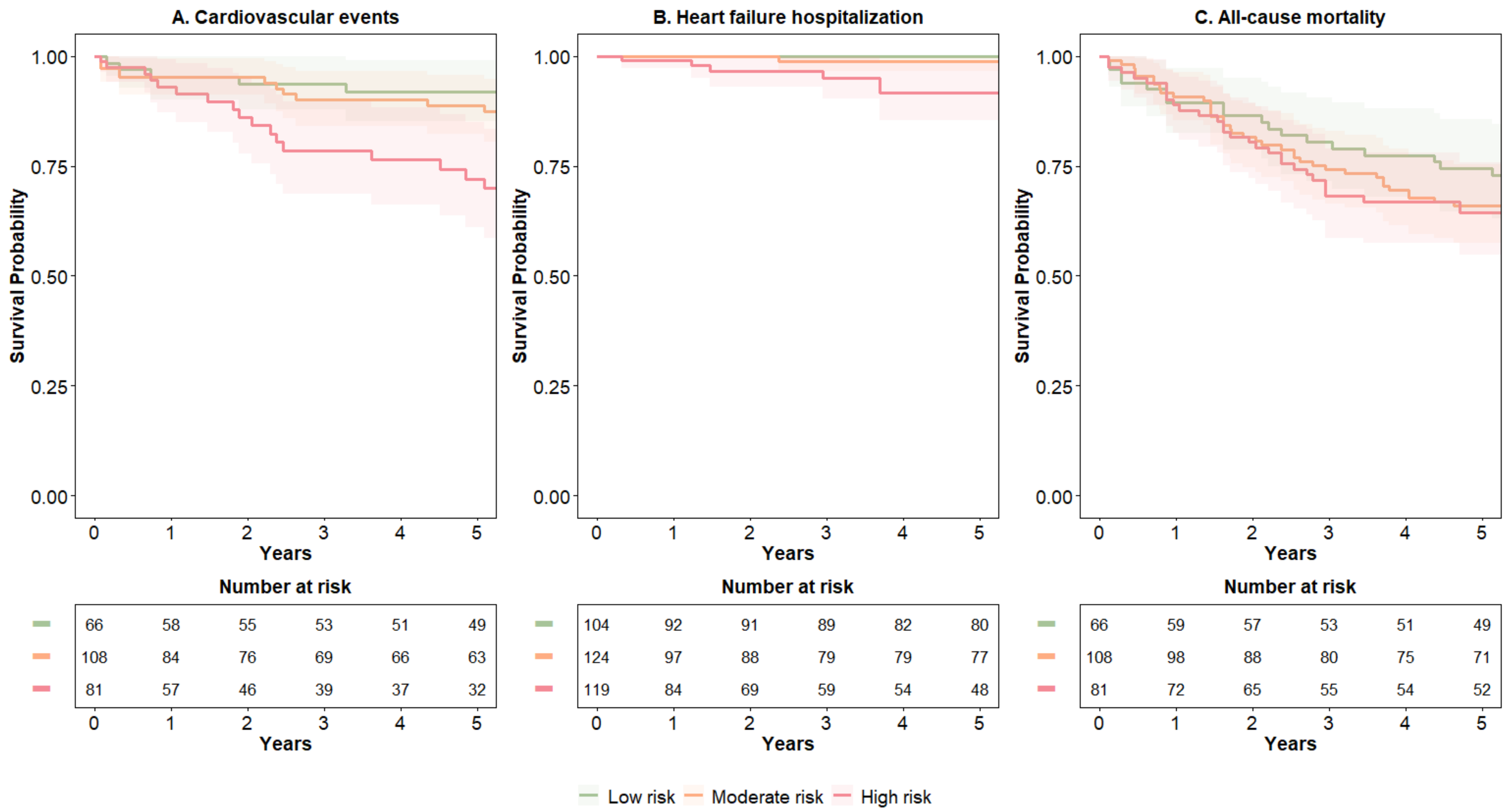

Figure S5. Survival curves for cardiovascular events (A), heart failure hospitalization (B) and all-cause mortality (C) among patients at low, moderate and high risk according to the HFA-ICOS risk stratification tool for patients receiving anthracyclines (n=255)

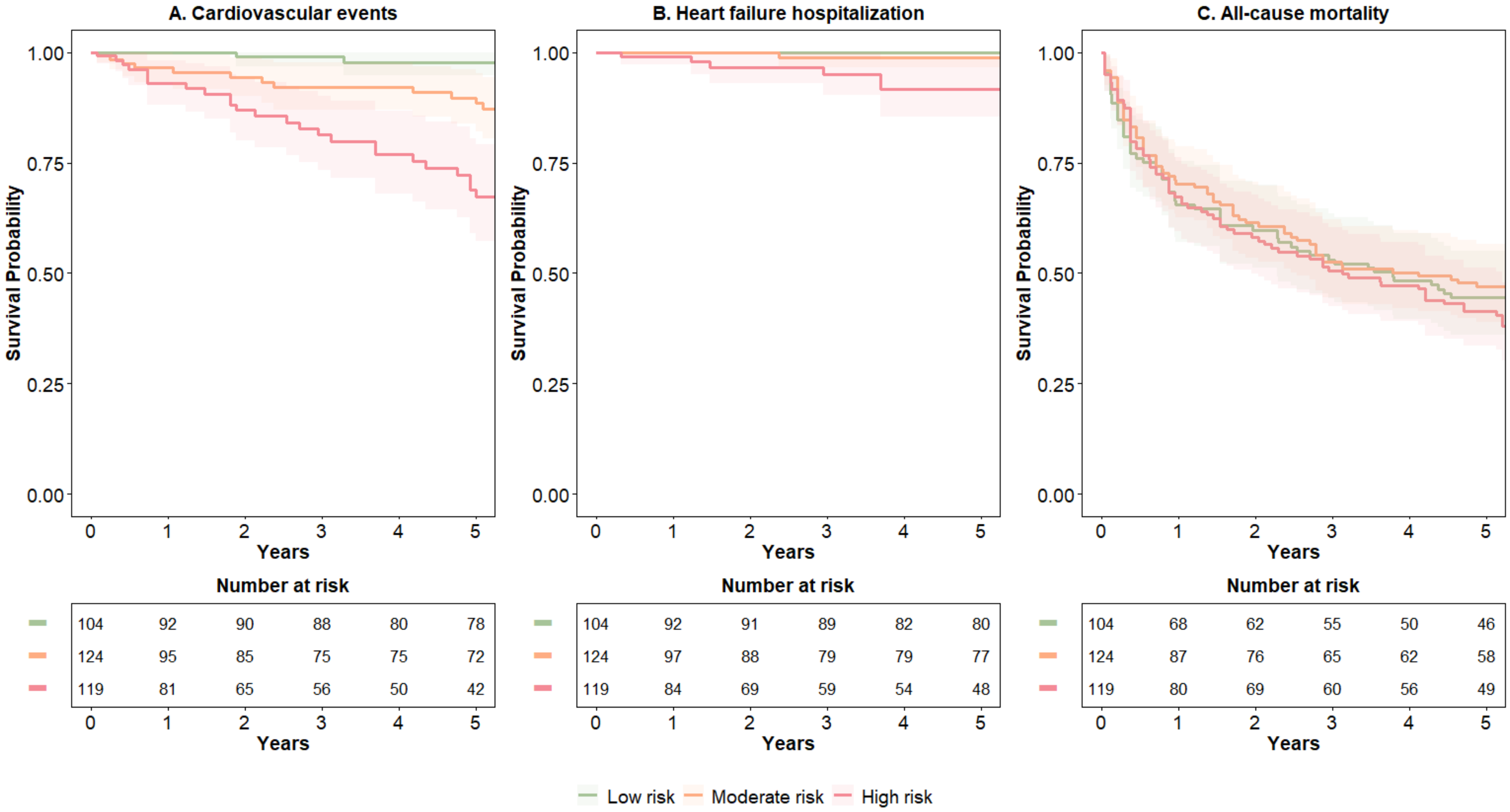

Figure S6. Survival curves for cardiovascular events (A), heart failure hospitalization (B) and all-cause mortalityS4 (C) among patients at low, moderate and high risk according to the HFA-ICOS risk stratification tool for patients receiving anthracyclines, HER2-targeted therapy, VEGF inhibitors or myeloma treatment (n=347).

References

1. Knopfholz J, Disserol CC, Pierin AJ, Schirr FL, Streisky L, Takito LL, et al. Validation of the friedewald formula in patients with metabolic syndrome. Cholesterol. 2014;2014:261878.
2. Govatsmark RES, Janszky I, Slørdahl SA, Ebbing M, Wiseth R, Grenne B, et al. Completeness and correctness of acute myocardial infarction diagnoses in a medical quality register and an administrative health register. Scand J Public Health. 2020;48(1):5-13.
3. Varmdal T, Bakken IJ, Janszky I, Wethal T, Ellekjaer H, Rohweder G, et al. Comparison of the validity of stroke diagnoses in a medical quality register and an administrative health register. Scand J Public Health. 2016;44(2):143-9.
4. Lyon AR, Dent S, Stanway S, Earl H, Brezden-Masley C, Cohen-Solal A, et al. Baseline cardiovascular risk assessment in cancer patients scheduled to receive cardiotoxic cancer therapies: a position statement and new risk assessment tools from the Cardio-Oncology Study Group of the Heart Failure Association of the European Society of Cardiology in collaboration with the International Cardio-Oncology Society. Eur J Heart Fail. 2020;22(11):1945-60.
5. Lyon AR, López-Fernández T, Couch LS, Asteggiano R, Aznar MC, Bergler-Klein J, et al. 2022 ESC Guidelines on cardio-oncology developed in collaboration with the European Hematology Association (EHA), the European Society for Therapeutic Radiology and Oncology (ESTRO) and the International Cardio-Oncology Society (IC-OS): Developed by the task force on cardio-oncology of the European Society of Cardiology (ESC). Eur Heart J. 2022;43(41):4229-361.
